# Experimental-Analytical Method for Temperature Determination in the Cutting Zone during Orthogonal Turning of GRADE 2 Titanium Alloy

**DOI:** 10.3390/ma14154328

**Published:** 2021-08-03

**Authors:** Łukasz Ślusarczyk

**Affiliations:** Chair of Production Engineering, Faculty of Mechanical Engineering, Cracow University of Technology, Al. Jana Pawła II 37, 31-864 Kraków, Poland; lukasz.slusarczyk@pk.edu.pl

**Keywords:** temperature, GRADE 2 titanium alloy, thermovision camera, Johnson-Cook model

## Abstract

The paper presents an experimental-analytical method for determination of temperature in the cutting zone during the orthogonal turning of GRADE 2 titanium alloy. A cutting insert with a complex rake geometry was used in the experiments. The experimental part of the method involved orthogonal turning tests during which the cutting forces and the chip forming process were recorded for two different insert rake faces. The analytical part used a relationship between the cutting forces and the temperature in the Primary Shear Zone (PSZ) and the Secondary Shear Zone (SSZ), which are described by the Johnson-Cook (J-C) constitutive model and the chip forming model according to the Oxley’s theory. The temperature in the PSZ and SSZ was determined by finding the minimum difference between the shear flow stress determined in the J-C model and the Oxley’s model. Finally, using the described method, the relationship between the temperature in the PSZ and SSZ and the rake face geometry was determined. In addition, the temperature in the cutting zone was measured during the experimental tests with the use of a thermovision camera. The temperature distribution results determined experimentally with a thermovision camera were compared with the results obtained with the described method.

## 1. Introduction

Machining is one of the most important technologies of making the parts of machines and equipment. An intensive development of this production technology forces a rationalization of the factors which could reduce the costs. One problem during the tool selection is increasing or keeping its life when the operating conditions change. In this case, the problem of high temperatures in the cutting zone that adversely affect the tool life and wear is very important [1,2]. The experimental and analytical methods for temperature determination in the cutting zone are widely described in the literature [3,4,5,6,7]. However, there is no one specific optimal method. Depending on the studies conducted, one expects the ability to measure the average and maximum temperature at the chip-edge contact area, and also the temperature distribution at the chip-edge contact area, as well as the temperature distribution at the contact length or in the entire heat flow zone. The experimental temperature measurement methods include the conduction- and radiation-based methods. The conduction-based methods were described by Davies et al. and Nedić et al. [8,9]. One of them is the Resistance Method (RM) which involves the change of the sensor resistance along with the change of temperature. The Resistance Thermometry Devices (RTD) are often made of pure platinum and they can be implemented as microthermistors on the cutting tool rake face. The shortcoming of this method is low resistance of the sensors to damage and high susceptibility to interference which occurs during the machining. The other method is Thermocouple (TC/DTC) which allows a point temperature measurement on the rake face. This method involves the use of inserts of foreign thermocouples and the Seebeck effect. The advantage of this method, when thermocouples of known characteristics are used, lies in obtaining direct measurements in temperature units, but the problem lies in the need to make precision, thin holes in the hard tool material. This is connected to the weakening of the tool structure which limits the method application to a narrow range of machining parameters. The obtained temperature values are average values. Other experimental methods are based on the Thermophysical Processes (TP). They are classified as semi-invasive and include thermo-sensitive and thermo-indicating paints, thermo-chromatic liquid crystals, thermographic phosphors, temperature sensitive crayons and pellets and pyrometric cones. These methods are used rather seldom and have high measurement uncertainty. The temperature measurement can be performed after completion of the process. Other interesting methods of thermocouple implementation can be found in the literature, as well. Sugita et al. described the proposal of temperature sensor built into the cutting tool as a sensor array on the rake face of the cutting tool [10]. The paper describes an array composed of 10 sensors durably placed on the rake face. The experiment involved machining of a shaft made of non-conducting nylon. The authors emphasize that this solution can be used in the machining of medical materials. Basti et al. presented an interesting temperature measurement technique [11]. The photolithography was used to make thin structures which are Ni-Cr thermocouples (Thin Film Thermocouples—TFTs) protected against the chip by the TiC or TiAlN/TTT layers. Such prepared tools allow the temperature measurement in selected points on the rake face. The temperatures generated in the edge protective layers are obtained. These methods allow obtaining information on the temperature distribution on the cutting insert rake face. However, their implementation is very complicated and practically precludes their use in the industry [12,13]. The experimental techniques based on the heat radiation are becoming increasingly important. Depending on the type and method of machining, the measurement setups are built with the use of pyrometers or thermovision cameras suitably adapted to the expectations of the researchers [14,15,16,17,18]. The measurement is contactless and the recorded radiation spectrum allows determining the average and maximum point temperature values, as well as the temperature fields in the cutting zones. Arrazola et al. published the results of contact temperature measurements with the use of thermovision camera during orthogonal turning [19]. The authors indicate the limitation of the described method. Placing the thermovision camera sideways to the cutting zone causes the measurement of the temperature on the lateral part of the chip. According to the authors, depending on the machined material and the cutting parameters, the difference of contact temperature (in the SSZ) is from 60 to 290 °C. Davies et al. described the use of a thermovision camera during orthogonal turning of the AlSl 1045 steel [20]. The temperature measurements in the SSZ were made for different chip thickness. The obtained results were used for verification of the temperature calculations with the analytical methods. Abouridouane et al. described the use of a high-speed thermovision camera (328 fps) to monitor the cutting zone temperature during the cutting of AISI 1040 and AISI 4140 steel specimens [21]. Analytical calculation models are a separate group of temperature determination methods in the cutting zone. They are based on the relationships from different fields of science (materials science, physics) applied to describe the material behaviour during decohesion. In a particular case of temperature models of orthogonal turning, the temperature increase in two zones is studied: In the primary plastic strain zone, in which the main plastic strain mechanism is slip, and in the secondary plastic strain zone where the chip friction against the tool rake face also becomes important [22]. The majority of the model-based (J-C, Oxley’s) analytical methods use the assumptions: 1—cutting tool is perfectly sharp and not worn, 2—chip forming process is continuous, 3—the heat flux loss as a result of convection and radiation from the surface of the workpiece, tool, and chip are negligible, 4—chip thickness over the entire contact area with the rake face is constant, 5—the directions of slip forces and of the force tangential to the tool rake face are collinear to the respective directions of the slip and chip speed vectors. Adequate knowledge of the contact temperature in the cutting zone at specific conditions is an important element in determining the thermo-mechanical cutting process [23,24,25,26,27]. The use of analytical method for determination of temperature in the SSZ was presented by Chen et al. [28]. The innovation described in the paper is the heat source modelling. A uniformly distributed rectangle near the cutting edge is used rather than a flat heat source. This approach is based on Oxley’s model and the J-C constitutive equation. The validation of the analytical calculation method for variable cutting parameters (cutting speed *V* and feedrate *f* and also variable tool rake angle (*α*) is performed for the experimental data of turning the AISI 1045 steel, Al 6086-T6, and Ti6Al4V. The use of Oxley’s model and the J-C equation for the identification of the temperature distribution in the cutting zone was also described by Aydin [29]. The author’s approach is based on a thermal model accounting for the impact of the primary and secondary heat source. The proposed model was successfully verified with experimental results of machining the AISI 1045 steel. The use of numerical methods for temperature determination in the cutting zone has been described in [30,31]. Umbrello et al. used the FEM to simulate turning of Ti-6Al-4V and analyses cutting forces and chip morphology [32]. Liu used the FEM to determine the residual stresses in the machined layer [33]. List et al. determined the temperature in the cutting zone based on the developed numerical model which accounts for crater wear on the tool face [34].

## 2. Methodology

The paper presents an experimental-analytical method for temperature determination in the PSZ and SZZ during orthogonal turning. The assumptions in the method are that the orthogonal cutting process is stabilized and the tool is perfectly sharp and unworn. The method is based on the approach proposed by Ning et al. in [35]. However, this approach has been modified by adding certain original solutions. In the experimental part, the cutting forces *F_c_* and *F_f_*, shear angle *ϕ*, and the shear line length *l_AB_* were determined. During experimental tests, the cutting forces were determined using a piezoelectric dynamometer. However, the shear angle *ϕ* and the shear line length *l_AB_* were determined experimentally using a proprietary stand for taking photographs of the chip formed during the cutting process. At the next stage, the photographs were analysed using a specialist software to determine the actual values of shear angle *ϕ* and the shear line length *l_AB_*. The values determined experimentally were used further in the paper. The method is based on the J-C constitutive (equation) model and the cutting mechanics described by the Oxley model. The J-C constitutive equation is used to describe the stress-strain relation in the machined material in which high intensity plastic strain occur:

(1) $$\sigma =\left(A+B\varepsilon^{n}\right)\left[1+Cln\left(\frac{\dot{\varepsilon }}{{\dot{\varepsilon }}_{0}}\right)\right]\left[1-{\left(\frac{T-T_{r}}{T_{m}-T_{r}}\right)}^{m}\right]$$

where *σ* is the material flow stress, *ε* is the plastic strain, $\dot{\varepsilon }$ is the plastic strain rate,$\dot{\;\varepsilon_{0}}\;$ is the reference plastic strain rate, *T* is the temperature of the workpiece material, *T_r_* is the reference temperature, and *T_m_* is the melting temperature of material. *A*, *B*, *C*, *m*, and *n* are five Johnson–Cook material parameters (J-C constants). *A* and *B* are the strain hardening coefficients, *C* is the dimensionless strain-rate hardening coefficient, *m* and *n* are the exponential terms of the strain hardening term and thermal softening term, respectively. The mean temperature in the PSZ (*T_AB_*) has been determined by finding the minimum difference between the shear stress determined in the mechanical model (*k_AB_*) and the J-C model (*k’_AB_*).

(2) $$k_{AB}=\frac{F_{s}}{l_{AB}w}$$

(3) $${k^{\prime }}_{AB}=\frac{\sigma_{AB}}{\sqrt{3}}=\frac{1}{\sqrt{3}}\left(A+B\varepsilon^{n}{}_{AB}\right)\left(1+Cln\frac{{\dot{\varepsilon }}_{AB}}{\dot{\varepsilon_{0}}}\right)\left(1-{\left(\frac{T_{AB}-T_{r}}{T_{m}-T_{r}}\right)}^{m}\right)$$

In formula (2), the shear force (*F_s_*) is determined according to the following relationship:

(4) $$F_{s}=Rcos\left(\phi +\lambda -\alpha \right)$$

where in *l_AB_*, the primary shear zone length and the shear angle *ϕ* have been determined experimentally and *w* is the cutting width (depth). The friction angle *λ* has been determined according to the following relationship:

(5) $$\lambda =atan\left(\frac{F_{t}}{F_{c}}\right)+a$$

and in formula (3), the strain $\varepsilon_{AB}$ and strain rates $\dot{\varepsilon_{AB}}$ have been determined according to the following relationship:

(6) $$\varepsilon_{AB}=\frac{\gamma_{AB}}{\sqrt{3}}=\frac{cos\alpha }{2\sqrt{3}sin\phi cos\left(\phi -\alpha \right)}$$

(7) $$\dot{\varepsilon_{AB}}=\frac{\dot{\gamma_{AB}}}{\sqrt{3}}=C_{0}\frac{V_{s}}{\sqrt{3}l_{AB}}$$

where *C*_0_ is the strain rate constant and *V_s_* is the shear velocity:

(8) $$V_{s}=\frac{Vcos\alpha }{cos\left(\phi -\alpha \right)}$$

The mean temperature in the SSZ (*T_int_*) has been determined by finding the minimum difference between the shear flow stress determined in the mechanical model (*τ_int_*) and the J-C model (*k_int_*):

(9) $$\tau_{int}=\frac{F}{hw}$$

(10) $$k_{int}=\frac{1}{\sqrt{3}}\left(A+B\varepsilon^{n}{}_{int}\right)\left(1+Cln\frac{\dot{\varepsilon_{int}}}{\dot{\varepsilon_{0}}}\right)\left(1-{\left(\frac{T_{int}-T_{r}}{T_{m}-T_{r}}\right)}^{m}\right)$$

where *F* is the shear force at the PSZ, *R* is the resultant force, and *h* is the tool-chip interface.

(11) F=Rsin(λ)

(12) $$R=\frac{F_{c}}{cos\left(\lambda -\alpha \right)}$$

*h* is the tool-chip interface:

(13) $$h=\frac{t_{1}sin\theta }{cos\lambda sin\phi }\left(1+\frac{C_{0}n_{eq}}{3\left(1+2\left(\frac{\pi }{4}-\phi \right)-C_{0}n_{eq}\right)}\right)$$

(14) $$n_{eq}\approx \frac{nB\varepsilon^{n}{}_{AB}}{\left(A+B\varepsilon^{n}{}_{AB}\right)}$$

and in formula (10), the reference strain rate $\dot{\varepsilon_{0}}$ = 1, the strain rate constant δ, the strain $\varepsilon_{int}$, and strain rates $\dot{\varepsilon_{int}}$ have been determined according to the following relationship:

(15) $$\varepsilon_{int}=\frac{\gamma_{int}}{\sqrt{3}}=2\varepsilon_{AB}+\frac{h}{2\sqrt{3}\delta t_{2}}$$

(16) $$\dot{\varepsilon_{int}}=\frac{\dot{\gamma_{int}}}{\sqrt{3}}=\frac{V_{c}}{\sqrt{3}\delta t_{2}}$$

**Figure 1:**
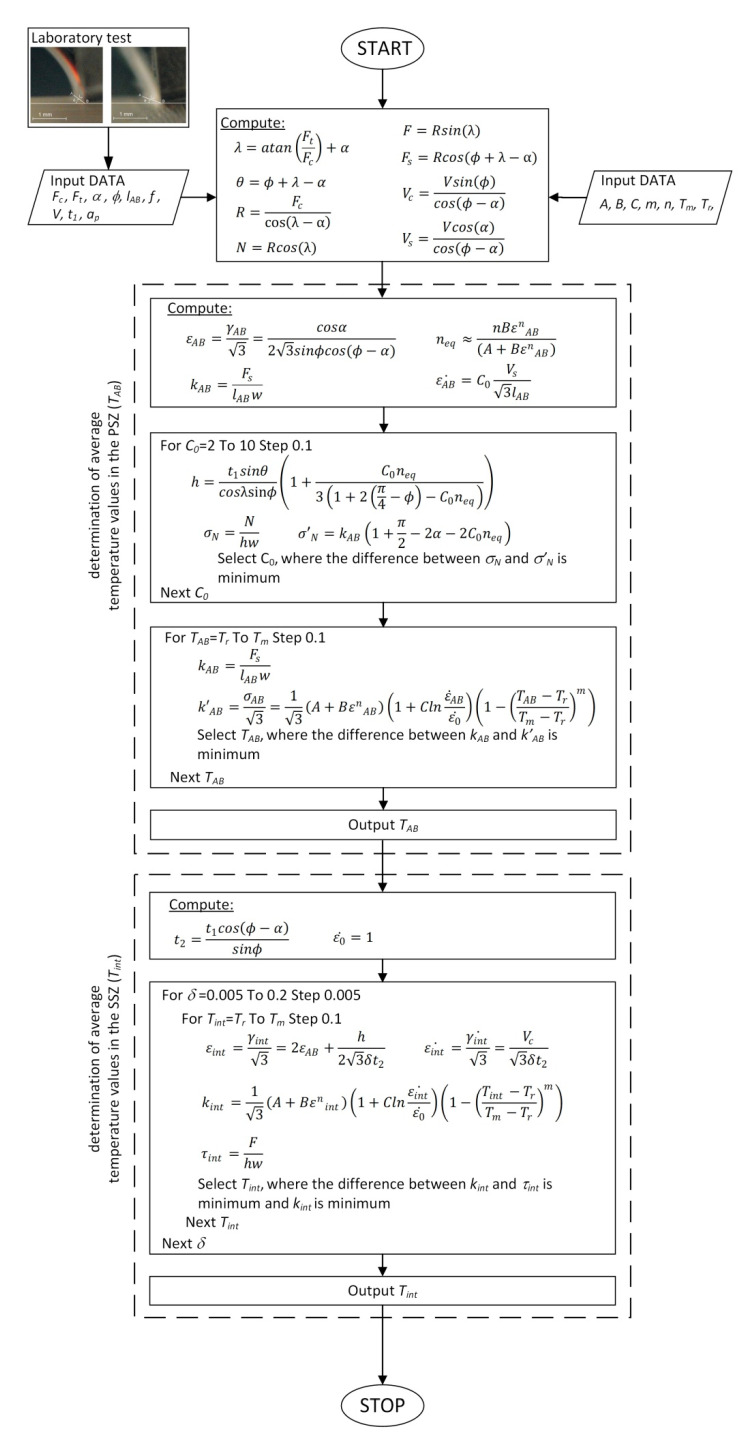
Algorithm for determination of average temperature values in the PSZ and SSZ.

The method for temperature determination in the PSZ and SSZ is based on the algorithm presented in Figure 1. The input parameters in the algorithm are the experimentally determined cutting forces: *F_c_*, *F_t_*, shear angle: φ and the shear zone length *l_AB_* and, in addition, the cutting depth *w,* and constants for the J-C equation. The first algorithm step involved the determination of constant *C_0_*. This includes the recurrent determination and comparison of normal stress at the SSZ for the mechanical model $\sigma_{N}$ and J-C ${\sigma^{\prime }}_{N}$. The selection of *C*_0_ values takes place for the minimum difference $\sigma_{N}$ and ${\sigma^{\prime }}_{N}$.

(17) $$\sigma_{N}=\frac{N}{hw}$$

(18) $${\sigma^{\prime }}_{N}=k_{AB}\left(1+\frac{\pi }{2}-2\alpha -2C_{0}n_{eq}\right)$$

The second algorithm step involves the temperature determination in the PSZ (*T_AB_*). The *T_AB_* value in the range from *T_r_* to *T_m_* is changed iteratively. The values of shear flow stress *k_AB_* and *k’_AB_* are compared at each iteration. Temperature *T_AB_* is determined for the least difference between the shear flow stress. The temperature in the SSZ in the third algorithm step is determined in an analogous manner. The difference between *τ_int_* and *k_int_* is determined for recurrently changing *T_int_*. The *T_int_* is selected for the least difference between them. The algorithm has been implemented in the Visual Basic language in the MS EXCEL 2019 environment. The algorithm for determination of the mean temperature values in the presented method is shown in Figure 1. The described algorithm is a part of the proposed method which allows calculating the average temperature in the PSZ and SSZ using the correlation between the input data and the temperature. The method is rather not complex experimentally since the input data were obtained during the laboratory tests. These data include the values of cutting forces which were measured with a triaxial piezoelectric dynamometer, and the shear angle *ϕ*, as well as the shear line length *l_AB_*. The method of determining the experimental input data was described further in the paper. The method does not require temperature-sensitive material data such as thermal conductivity or specific heat of the machined material. However, the method has its limitations since the quality of prediction of the mean temperature values in the PSZ and SSZ depends on the accurate experimental data: Chip thickness, cutting forces, shear angle, shear line length, and the J-C equation constants. The validation of the temperature values obtained using the described method with the values obtained using a thermovision camera during the laboratory tests is described further in the paper.

## 3. Materials and Methods

### 3.1. Material

The laboratory experiments involved a series of orthogonal turning tests of D = 60 mm tube made of GRADE 2 titanium alloy. The chemical composition of the GRADE 2 titanium alloy, in accordance with the EN 10204-3.1 standard, is presented in Table 1.

Some properties of GRADE 2 titanium alloy are presented in Table 2.

### 3.2. Methods

**Figure 2:**
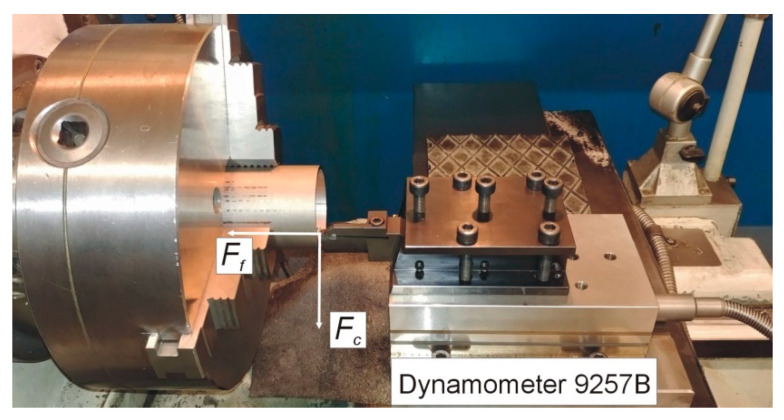
Dynamometer fixing and distribution of forces during the orthogonal turning [36].

The laboratory setup was equipped with a KNUTH Masterturn 400 precision lathe and the measurement rig for recording the cutting forces. The measurement rig comprised a 9257B piezoelectric dynamometer placed on the lathe carriage and a 5070B charge amplifier from Kistler (Winterthur, Switzerland). The signal representing the cutting force components was recorded with the 1000 Hz frequency, and the received values were analysed using the DynoWare software (version 2825A, Kistler Group, Winterthur, Switzerland). The dynamometer fixing in the lathe carriage and the distribution of forces during the orthogonal turning is presented in Figure 2.

**Figure 3:**
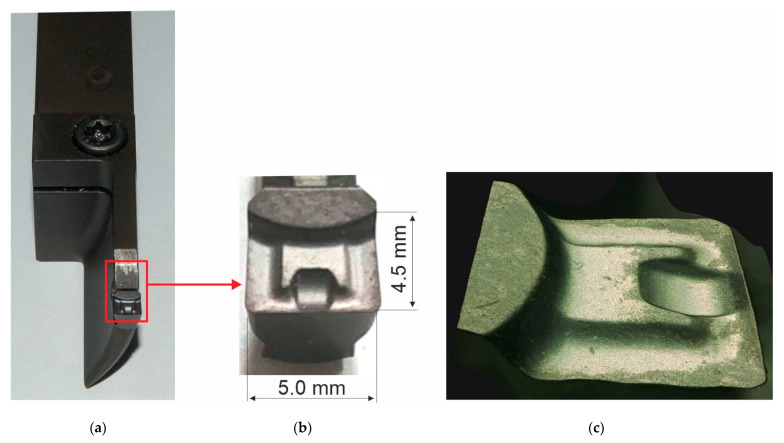
(**a**) Kennametal tool; (**b**) top and dimensions; (**c**) side views of the rake face [36].

The tests were made with the use of a Kennametal A3G0500M05P04DF insert (Kennametal Inc., Pittsburgh, PA, USA). The insert was placed in a A3SAR2520M0530-075-100 holder (Kennametal Inc., Pittsburgh, PA, USA). The insert rake face is presented in Figure 3.

**Figure 4:**
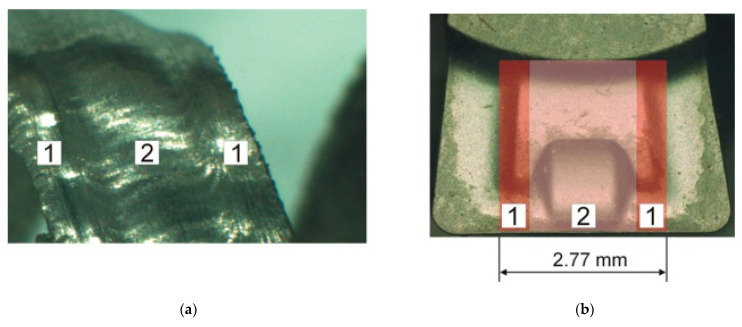
(**a**) Characteristic areas 1 and 2 on the chip surface and (**b**) corresponding areas on the rake face of the insert [36].

The initial experimental orthogonal turning tests for the full tube thickness, *a_p_* = 2.77 mm, indicated a characteristic form of the chip inside the surface related to different chip flow speeds on the rake face. The areas 1 and 2 on the chip and on the tool rake face were measured and determined experimentally. The width of area 1 on the insert face was 0.5 mm, and the width of area 2 was 1.77 mm, (Figure 4). 

**Figure 5:**
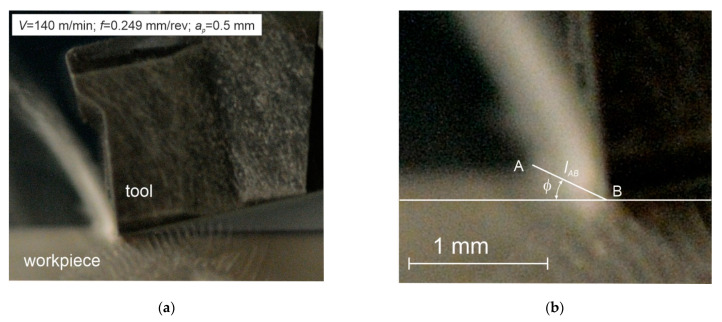
(**a**,**b**) Examples of the chip forming photographs for *V* = 140 m/min, *f* = 0.249 mm/rev, *a_p_* = 0.5 mm.

**Figure 6:**
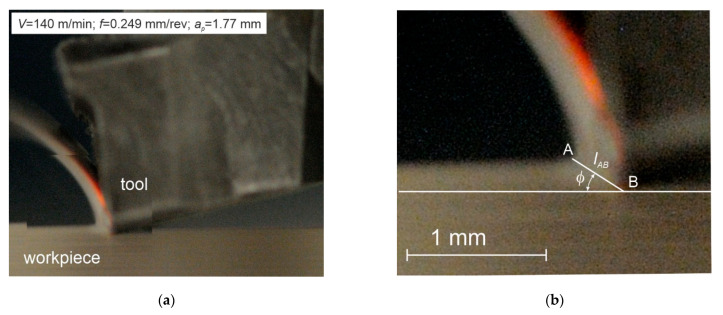
(**a**,**b**) Examples of the chip forming photographs for *V* = 140 m/min, *f* = 0.249 mm/rev, *a_p_* = 1.77 mm.

The proper experimental studies required the tube wall thickness reduction and involved two series of orthogonal turning. The first series was performed for the tube wall thickness *a_p_* = 0.5 mm, where the chip is formed by area 1 on the insert rake face. The second series was performed for the tube wall thickness of *a_p_* = 1.77 mm, where the chip is formed by area No. 2. The cutting force components, *F_c_* (tangential component) and *F_f_* (feed component), were determined experimentally during each series. In addition, the chip photographs were taken during the tests for the cutting depth of *a_p_* = 0.5 mm and *a_p_* = 1.77 mm. The camera was a Nikon D300s with a Sigma f 2.8 EX MACRO 18–50 mm lens, in the serial photographs mode. Examples of the photographs are shown in Figure 5 and Figure 6. The photographs were used in the graphic software to determine the rake angle φ and the cutting zone length *l_AB_*.

In addition, the rake angle was measured experimentally in the tool system of coordinates, in area 1 and 2 using a Taylor Hobson Intra 50 profilographometer (Rank Taylor Hobson Ltd. Leicester, UK). The average rake angles in the tool system of coordinates were *α* = +7° for area 1 and *α* = +15° for area 2. These values were used in further calculations. 

### 3.3. Experiment Details

The plan of principal studies has been developed based on the Taguchi method. The independent variables were *V* and *f*. The parameters variability range was chosen on the basis of catalogue data and is presented in Table 3.

Each series for two different tube wall thicknesses included nine orthogonal turning tests, 18 tests in total. The parameter values for the individual tests are given in the Table 4 below.

**Figure 7:**
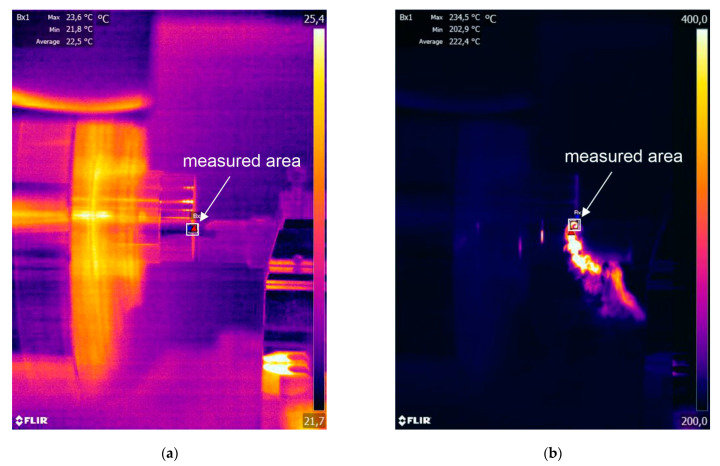
The thermovision image of the cutting zone (**a**) before the machining; (**b**) during the machining.

In addition, during the experiments the temperature distribution in the cutting zone was measured using a FLIR SC 620 (FLIR Company, Wilsonville, OR, USA) thermovision camera with an f = 38 mm prime lens. The recording frequency was 30 Hz, and the resolution was 640 ×480 px, the range from 0 to 500 °C was used in the tests. The minimum distance from the camera to the measured object that guaranteed obtaining a sharp image was about 80 cm. During the recording of thermovision sequences, in the limited marked area of the cutting zone, the side PSZ view and the view of the forming chip was dominated. The components of different emissivities occur in the marked, measured area, e.g., the workpiece, chip formed during the cutting process, cutting tool, a fragment of the tool holder. After preliminary trials and tests, the averaged emissivity value of ε = 0.8 was accepted to be used for the analysed area. The ambient temperature T_ref_ is 20 °C. The obtained thermograms have been analysed in the Therma Cam Researcher 2.9 application (FLIR Company, Wilsonville, OR, USA). The camera position allowed recording the temperature on the side of the chip flowing on the insert face. Figure 7 presents examples of thermovision images before and during the machining. In the marked limited area (square 5 × 5 mm) of the cutting zone recorded by the thermovision camera, the side PSZ view and a part of the forming chip was dominated. The maximum temperature in the PSZ was determined during the time-lapse analysis of thermovision images. The maximum average temperature value was determined for each trial. The measurements were conducted for all 18 orthogonal turning trials (Table 5). 

Further in the paper, the results obtained with the thermovision camera are compared with the temperature values determined analytically. In addition, the material constant for the J-C constitutive equation describing the machined GRADE 2 titanium alloy were used in the second, analytical part of the presented method (Table 6). Constants A, B, C, n, m have been taken from the literature [37]. 

## 4. Results

Table 7 includes experimentally determined mean cutting forces, where *F_f_* is the feed component and *F_c_* is the tangential component for tube wall thickness *a_p_* = 0.5 mm and *a_p_* = 1.77 mm (zones 1 and 2). 

**Figure 8:**
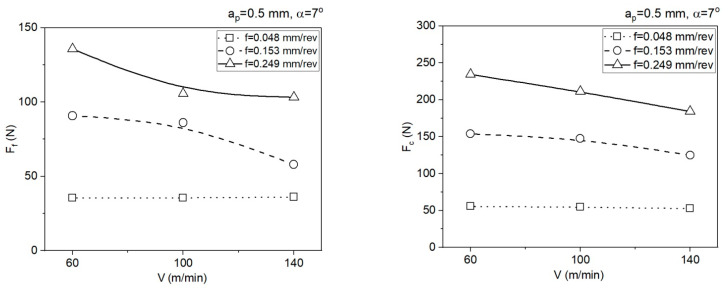
Impact of the cutting data on the values of the cutting force components *F_f_* and *F_c_*, *a_p_* = 0.5 mm, *α* = 7°.

**Figure 9:**
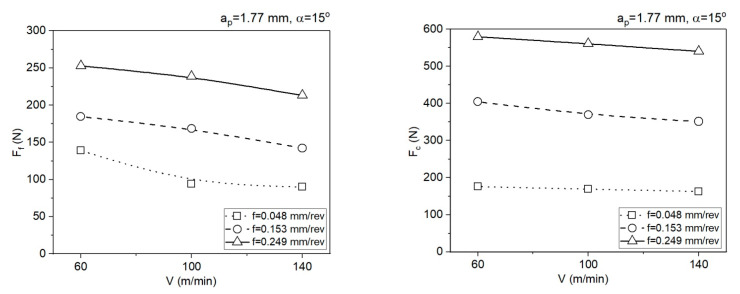
Impact of the cutting data on the values of the cutting force components *F_f_* and *F_c_*, *a_p_* = 1.77 mm, *α* = 15°.

Figure 8 and Figure 9 below present the impact of cutting parameters *V* and *f* on the mean cutting forces *F_f_* and *F_c_* for area 1 (*a_p_* = 0.5 mm) and area 2 (*a_p_* = 1.77 mm).

Table 8 presents the experimentally determined values of shear angle *φ* and the chip-edge contact length *l**_AB._***

Table 9, Table 10, Table 11 and Table 12 include the values determined in the analytical part of the presented method.

**Figure 10:**
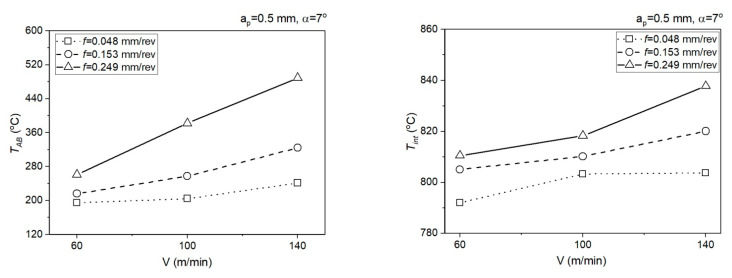
Impact of the cutting data on the mean temperature in the PSZ and SSZ, *a_p_* = 0.5 mm, *α* = 7°.

Figure 10 presents the relationship of analytically determined mean temperature in the PSZ (*T_AB_*) and SSZ (*T_int_*) for *a_p_* = 0.5 mm. 

**Figure 11:**
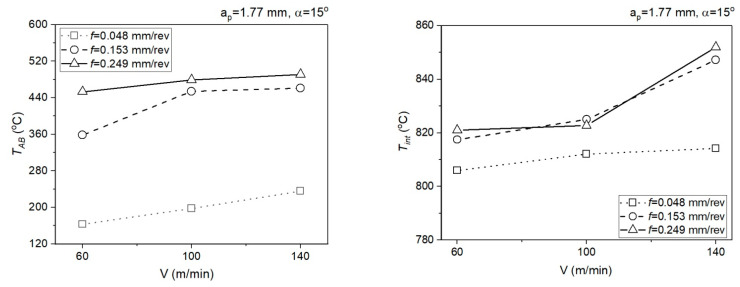
Impact of the cutting data on the mean temperature in the PSZ and SSZ, *a_p_* = 1.77 mm, *α* = 15°.

Figure 11 presents the relationship of analytically determined mean temperature in the PSZ (*T_AB_*) and SSZ (*T_int_*) for *a_p_* = 1.77 mm.

**Figure 12:**
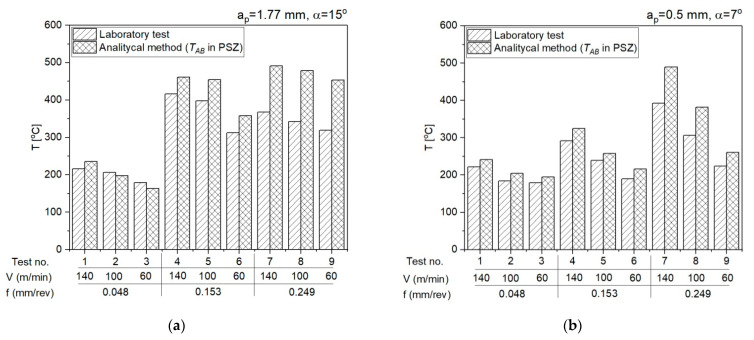
The mean temperature values in the PSZ obtained using the analytical method and the results of measurements with a thermovision camera. (**a**) *a_p_* = 1.77 mm, *α* = 15°; (**b**) *a_p_* = 0.5 mm, *α* = 7°.

The mean temperature values in the PSZ obtained using the analytical method and the results of measurements with a thermovision camera during the laboratory tests are presented below in Figure 12.

**Figure 13:**
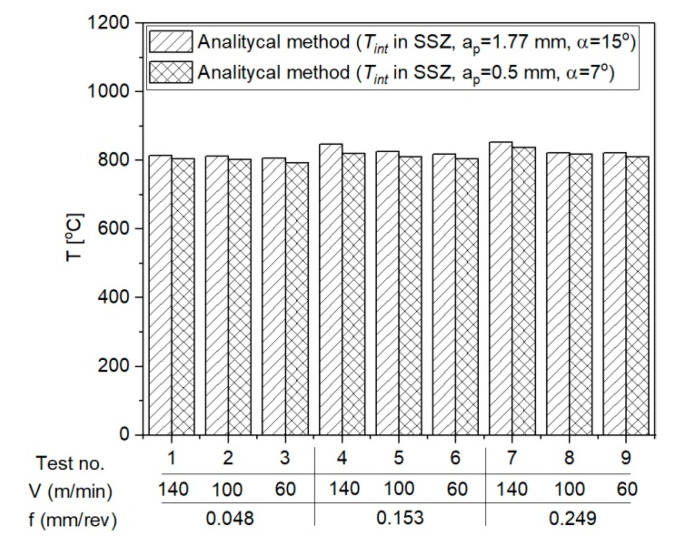
The mean temperatures in the SSZ obtained with the use of analytical method for two different cutting depths, *a_p_* = 1.77 mm and *a_p_* = 0.5 mm.

Figure 13 presents the mean temperature in the SSZ obtained with the use of analytical method for two different cutting depths. 

**Figure 14:**
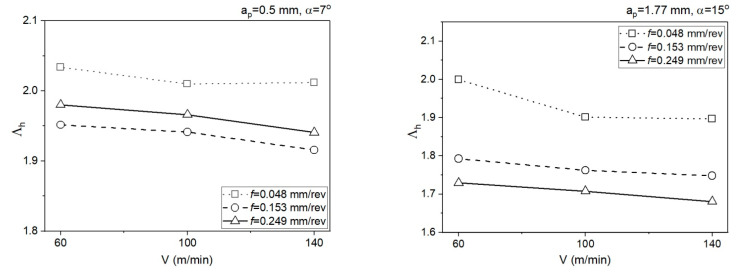
Impact of the cutting data on the values of the chip compression ratio for SSZ.

Figure 14 presents the relationship of the chip compression ratio for SSZ. The chip compression ratio was determined from relationship (19):

(19) $$\Lambda_{h}=\frac{V}{V_{c}}$$

**Figure 15:**
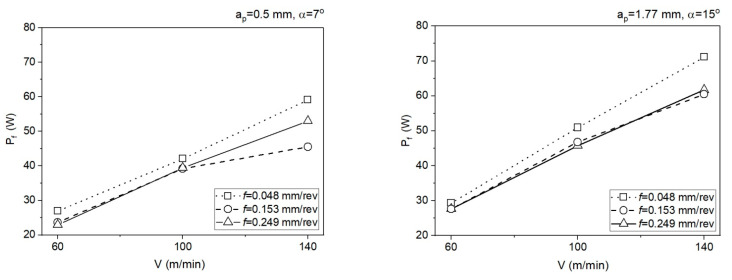
The friction power determined in the SSZ.

The friction power determined in the SSZ for *a_p_* = 0.5 mm and *a_p_* = 1.77 mm is presented below (Figure 15). The friction power was determined from relationship (20):

(20) $$P_{f}\;=\;FV_{c}$$

**Figure 16:**
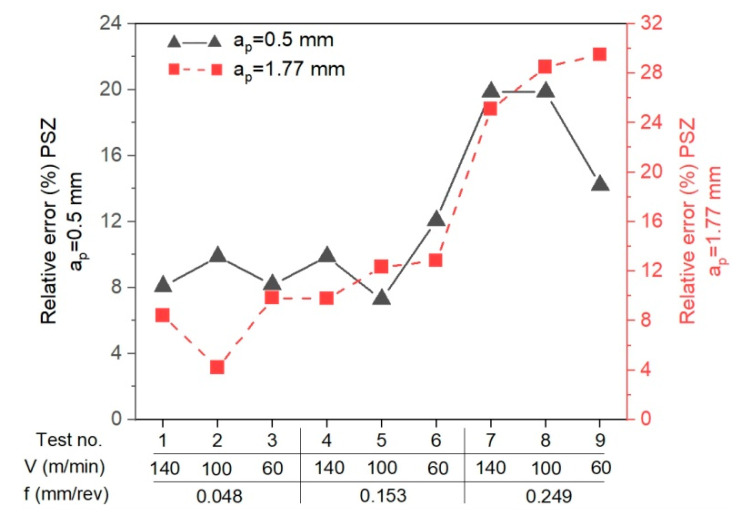
The relative error between the results from calculations and the results from measurements with the thermovision camera for each test.

The relative error for the results from the analytical method and the results from the IR measurements was determined according to the formula D = abs(T_AB_ − T_ref_)/T_ref_, where T_AB_ is the mean temperature in the PSZ determined analytically and T_ref_ is the mean temperature in the PSZ determined experimentally with a thermovision camera. The verification of results obtained analytically in PSZ with the measurements conducted with the thermovision camera indicates the conformity of results for smaller feedrates (f = 0.048 mm/rev, f = 0.153 mm/rev). The maximum relative error between the results from calculations and the results from measurements with the thermovision camera is 13%. For feedrate f = 0.249 mm/rev, the error is greater and equal to 30% (Figure 16). The greater difference between the calculated temperature and the measured temperature results probably from the larger cross-section of the cutting zone and the larger chip heat capacity.

## 5. Conclusions

The paper presents a proprietary method for determination of the temperature in the cutting zone during the orthogonal turning. The advantages of the proposed method include the lesser experimental complexity, lesser mathematical complexity, and high calculation capacity. As a result, it can be a less expensive alternative to other methods of temperature determination in the cutting zone (thermovision cameras, thermocouples inserted into the cutting inserts or numerical simulations which are complex and difficult to implement). The experimental-analytical method was used to determine the mean temperature values in the PSZ and SSZ for two areas of cutting insert rake faces. Area 1 corresponds to the rake face of width a_p_ = 0.5 mm and has the +7° tool rake angle, while area 2 is located in the central part of the rake face, has the width of a_p_ = 1.77 mm and the +15° tool rake angle. The input parameters in the method are the experimentally determined cutting forces and additionally experimentally measured values of shear angle and the shear zone length. Normal and tangential stresses were determined for the PSZ and SSZ based on two different models: Oxley’s cutting mechanics model and the J-C model. The temperatures in the PSZ and SSZ were estimated by the recurrent determination of the minimum value between stresses obtained for two different models. In the presented method, a part of the algorithms for temperature determination in the PSZ will be more sensitive to the values of input parameters (cutting forces, tool geometry) than the part of the algorithm for temperature determination in the SSZ. The advantage of the proposed method is that it does not require temperature-sensitive material data such as thermal conductivity or specific heat of the machined material. However, its main limitation is that it gives one, mean temperature value in the PSZ and SSZ and that the determined temperature strictly depends on the input data.

- The calculation algorithm for determination of the mean temperature in the PSZ and SSZ was implemented in the VBasic MS EXCEL environment. The mean temperature values in the PSZ determined using the developed method correspond to the experimental results. The verification of results obtained analytically with the measurements conducted with the thermovision camera indicates the conformity of results for smaller feedrates (*f* = 0.048 mm/rev and *f* = 0.153 mm/rev). The maximum relative error between the results from calculations and the results from measurements with the thermovision camera in PSZ is 13%. For feedrate *f* = 0.249 mm/rev, the maximum error is greater and equal to 29%. Probably, the greater difference between the calculated temperature and the measured temperature results from the larger cross-section of the cutting zone and the larger chip heat capacity.
- The different shapes of cutting insert face in the two analysed areas translate to different chip flow speeds *V_c_* and chip compression ratios. In area 1 (*a_p_* = 0.5 mm, *α* = 7°), the chip compression ratio is greater in area 2 (*a_p_* = 1.77 mm, *α* = 15°). The increase of the chip compression ratio in area 1 relative to area 2 is 1.2–15.5%, depending on the feedrate. Undoubtedly, the greater chip compression ratio in area 1 increases the stresses in this area in the PSZ and SSZ. The shear stresses in the PSZ in area 1 increase relative to area 2 by 1.9–26.2%, depending on the feedrate. In the SSZ, the shear stresses in area 1 increase relative to area 2 by 20.3–115.5%, but the increase of normal stresses is greater by 29.4–127.4%, depending on the feedrate.
- The correct selection of the J-C equation constants is important for obtaining the high degree of temperature prediction accuracy in the cutting zone. Several sets of constants available in the literature have been analysed in the course of the study, but only the set included in the paper was used in the presented method.
- The application of the method in two different areas of the cutting insert rake face allows a better understanding of the relationship between the cutting forces, insert face geometry, and the temperature values in the PSZ and SSZ.

## Figures and Tables

**Table 1:** The percentage chemical composition of GRADE 2 titanium alloy [36].

Symbol	Fe	C	N	O	H	Ti
GRADE 2 max	0.30	0.08	0.03	0.25	0.015	Bal

**Table 2:** The properties of GRADE 2 titanium alloy [36].

Melting Point (°C)	Density (kg × m^−3^)	Modulus of Elasticity (GPa)	Specific Heat Capacity (J × kg^−1^ × K^−1^)	Thermal Conductivity (W × m^−1^ × K^−1^)
ca. 1660	4510	105	526	16.4

**Table 3:** Values of cutting parameters [36].

Symbol	Cutting Parameters	Parameter Values		
A	*f* (mm/rev)	0.048	0.153	0.249
B	*V* (m/min)	60	100	140

**Table 4:** Parameter values for individual tests [36].

**Test No.**	**A**	**B**	***f* (mm/rev)**	***V* (m/min)**	***a_p_* (mm)**	***α* (degs)**
1	1	1	0.048	140	0.5	7
2	1	2	0.048	100	0.5	7
3	1	3	0.048	60	0.5	7
4	2	1	0.153	140	0.5	7
5	2	2	0.153	100	0.5	7
6	2	3	0.153	60	0.5	7
7	3	1	0.249	140	0.5	7
8	3	2	0.249	100	0.5	7
9	3	3	0.249	60	0.5	7
**Test No.**	**A**	**B**	***f* (mm/rev)**	***V* (m/min)**	***a_p_* (mm)**	***α* (degs)**
1	1	1	0.048	140	1.77	15
2	1	2	0.048	100	1.77	15
3	1	3	0.048	60	1.77	15
4	2	1	0.153	140	1.77	15
5	2	2	0.153	100	1.77	15
6	2	3	0.153	60	1.77	15
7	3	1	0.249	140	1.77	15
8	3	2	0.249	100	1.77	15
9	3	3	0.249	60	1.77	15

**Table 5:** The mean-maximum temperature during the stabilized machining.

Test	*a_p_* = 0.5 mm			*a_p_* = 1.77 mm		
	*f* (mm/rev)	*V* (m/min)	*T_max_avg_* (°C)	*f* (mm/rev)	*V* (m/min)	*T_max_avg_* (°C)
1	0.048	140	221.7	0.048	140	215.8
2	0.048	100	184.2	0.048	100	205.9
3	0.048	60	178.7	0.048	60	179.0
4	0.153	140	292.1	0.153	140	416.0
5	0.153	100	238.7	0.153	100	398.0
6	0.153	60	190.0	0.153	60	312.2
7	0.249	140	392.0	0.249	140	367.8
8	0.249	100	306.2	0.249	100	342.4
9	0.249	60	223.7	0.249	60	319.4

**Table 6:** Johnson-Cook model constants of GRADE 2 titanium alloy ($\dot{\varepsilon_{0}}$ = 1; *T_r_* = 23 °C).

Material	*A* (MPa)	*B* (MPa)	*C*	*m*	*n*
GRADE 2	390	815	0.0187	0.685	0.3

**Table 7:** Mean values of cutting forces recorded during the orthogonal turning of GRADE 2 titanium alloy.

Test	*a_p_* = 0.5 mm				*a_p_* = 1.77 mm			
	*f* (mm/rev)	*V* (m/min)	*F_f_mean_* (N)	*F_c_mean_* (N)	*f* (mm/rev)	*V* (m/min)	*F_f_mean_* (N)	*F_c_mean_* (N)
1	0.048	140	36.1	52.6	0.048	140	90.0	162.8
2	0.048	100	35.5	54.5	0.048	100	94.0	169.1
3	0.048	60	35.5	55.5	0.048	60	138.8	175.9
4	0.153	140	58.0	124.9	0.153	140	142.2	351.2
5	0.153	100	86.1	147.5	0.153	100	168.4	369.2
6	0.153	60	90.8	153.7	0.153	60	184.6	404.3
7	0.249	140	103.2	184.0	0.249	140	213.0	540.0
8	0.249	100	105.7	211.1	0.249	100	238.5	561.0
9	0.249	60	135.7	234.3	0.249	60	252.8	579.5

**Table 8:** Experimentally determined values of shear angle and shear zone length.

Test	*a_p_* = 0.5 mm				*a_p_* = 1.77 mm			
	*f* (mm/rev)	*V* (m/min)	*φ* (degs)	*l_AB_* (mm)	*f* (mm/rev)	*V* (m/min)	*φ* (degs)	*l_AB_* (mm)
1	0.048	140	27.76	0.103	0.048	140	30.53	0.094
2	0.048	100	27.60	0.104	0.048	100	30.46	0.095
3	0.048	60	28.69	0.105	0.048	60	29.03	0.099
4	0.153	140	30.24	0.304	0.153	140	32.97	0.281
5	0.153	100	29.86	0.307	0.153	100	32.72	0.283
6	0.153	60	29.71	0.309	0.153	60	32.20	0.287
7	0.249	140	29.87	0.500	0.249	140	34.20	0.443
8	0.249	100	29.50	0.506	0.249	100	33.70	0.449
9	0.249	60	29.30	0.509	0.249	60	33.30	0.454

**Table 9:** The values determined for *a_p_* = 0.5 mm, α = 7°.

Test	*f* (mm/rev)	*V* (m/min)	*T_AB_* (°C)	*T_int_* (°C)	*k_AB_* (MPa)	*k’_AB_* (MPa)	*k_int_* (MPa)	*τ_int_* (MPa)
1	0.048	140	241.2	803.7	577.01	576.95	276.56	276.55
2	0.048	100	204.4	803.3	596.97	565.38	291.29	291.25
3	0.048	60	194.6	792.0	598.88	598.89	308.31	308.29
4	0.153	140	324.1	820.1	518.07	518.09	130.89	130.82
5	0.153	100	257.5	810.2	553.53	553.55	165.30	165.33
6	0.153	60	216.1	805.1	573.31	573.33	185.34	185.33
7	0.249	140	489.1	837.8	432.67	432.67	129.73	129.71
8	0.249	100	382.1	818.3	481.88	481.86	166.44	166.51
9	0.249	60	260.7	810.5	542.12	542.10	193.74	193.72

**Table 10:** The values determined for *a_p_* = 0.5 mm, α = 7°.

Test	*f* (mm/rev)	*V* (m/min)	*C*_0_	*σ*’_*N*_ (N/mm^2^)	$\varepsilon_{AB}$	$\dot{\varepsilon_{AB}}$ (1/s)	$\varepsilon_{int}$	$\dot{\varepsilon_{int}}$ (1/s)
1	0.048	140	5.8	313.0	0.657	48,703	6.008	4070
2	0.048	100	5.8	328.8	0.660	33,266	5.919	2890
3	0.048	60	5.2	339.5	0.667	18,176	5.786	1708
4	0.153	140	9.4	210.1	0.619	27,101	7.139	1396
5	0.153	100	9.7	217.2	0.624	19,751	7.790	984
6	0.153	60	9.0	240.8	0.626	10,914	7.360	587
7	0.249	140	9.3	176.6	0.624	16,283	7.435	846
8	0.249	100	8.3	214.8	0.629	10,266	6.987	597
9	0.249	60	7.6	256.3	0.632	5602	6.565	355

**Table 11:** The values determined for *a_p_* = 1.77 mm, α = 15°.

Test	*f* (mm/rev)	*V* (m/min)	*T_AB_* (°C)	*T_int_* (°C)	*k_AB_* (MPa)	*k’_AB_* (MPa)	*k_int_* (MPa)	*τ_int_* (MPa)
1	0.048	140	235.6	814.2	565.10	565.08	207.05	207.05
2	0.048	100	197.6	812.1	585.40	585.42	216.45	216.45
3	0.048	60	163.0	806.0	504.62	504.59	256.21	256.17
4	0.153	140	461.0	847.2	436.58	436.61	89.65	89.63
5	0.153	100	454.0	825.0	438.30	438.30	92.82	92.91
6	0.153	60	358.2	817.5	479.62	479.64	134.50	134.49
7	0.249	140	491.0	852.0	416.91	416.89	83.82	84.23
8	0.249	100	478.8	822.7	421.33	421.30	87.15	87.17
9	0.249	60	453.0	821.0	430.47	430.47	89.67	89.87

**Table 12:** The values determined for *a_p_* = 1.77 mm, α = 15°.

Test	*f* (mm/rev)	*V* (m/min)	*C*_0_	*σ*’_*N*_ (N/mm^2^)	$\varepsilon_{AB}$	$\dot{\varepsilon_{AB}}$ (1/s)	$\varepsilon_{int}$	$\dot{\varepsilon_{int}}$ (1/s)
1	0.048	140	6.0	214.8	0.569	51,455	6.866	4070
2	0.048	100	5.9	223.6	0.570	36,359	6.839	2901
3	0.048	60	4.5	262.3	0.592	15,969	5.980	1668
4	0.153	140	8.8	119.2	0.538	25,836	9.041	1367
5	0.153	100	9.2	113.1	0.541	19,124	9.709	970
6	0.153	60	7.0	162.8	0.547	8649	7.511	573
7	0.249	140	7.7	132.4	0.525	14,370	9.251	867
8	0.249	100	8.2	124.9	0.530	10,823	9.527	611
9	0.249	60	8.6	120.7	0.534	6729	9.589	363

## Data Availability

The data presented in this study are available on request from the author. The data are not publicly available due to privacy.

## Nomenclature

PSZ	primary shear zone (with subscript AB)
SSZ	secondary shear zone or tool–chip interface (with subscript int)
*A*; *B*; *C*; *m*; *n*	yield strength; strength coefficient; strain rate coefficient; thermal softening coefficient; and strain hardening coefficient in the J–C model
*T_m_*; *T_r_*; *T*	melting temperature; room temperature; temperature
*T_AB_*; *T_int_*	calculated average temperature at the PSZ and SSZ
*V*; *V_c_*; *V_s_*	cutting velocity; chip velocity; shear velocity
*α*; *φ*; *λ*; *θ*	rake angle; shear angle; friction angle at the SSZ; angle between resultant force R and the PSZ
*w (a_p_*); *t_1_*; *t_2_*	width of cut; depth of cut; and chip thickness
*l_AB_*; *h*	length of the PSZ; the length of the SSZ (tool–chip contact length)
$\varepsilon_{AB}$; $\dot{\varepsilon_{AB}}$; $\varepsilon_{int}$; $\dot{\varepsilon_{int}}$	strains and strain rates at the PSZ and SSZ
$\dot{\varepsilon_{0}}$ = 1	reference strain rate
*C*_0_	Oxley’s constants (ratio of the shear plane length to the thickness of the PSZ)
*δ*	strain rate constant (ratio of the thickness of the SSZ to chip thickness)
*n_eq_*	strain hardening constant
*k’_AB_*	calculated shear stress at the PSZ using the J–C model
*k_AB_*	calculated shear stress at the PSZ using a mechanics model
*k_int_*	calculated shear stress at the SSZ using the J–C model
*τ_int_*	calculated shear stress at the SSZ using a mechanics model
*σ_N_*	calculated normal stress at the SSZ using a mechanics model
*σ’_N_*	calculated normal stress at the SSZ using the J–C model
*F_c_*	cutting force
*F_t_*	thrust force
*F_s_*	shear force at the PSZ
*N_s_*	normal force at the PSZ
*F*	shear force at the SSZ
*N*	normal force at the SSZ
*R*	resultant force
*P_f_*	friction power
$\Lambda_{h}$	chip compression ratio for SSZ
*D*	relative error for the results from the analytical method and the results from the IR measurements
